# Reinforcement Learning-Enabled Cross-Layer Optimization for Low-Power and Lossy Networks under Heterogeneous Traffic Patterns

**DOI:** 10.3390/s20154158

**Published:** 2020-07-26

**Authors:** Arslan Musaddiq, Zulqar Nain, Yazdan Ahmad Qadri, Rashid Ali, Sung Won Kim

**Affiliations:** 1 Department of Information and Communication Engineering, Yeungnam University, 280 Daehak-Ro, Gyeongsan, Gyeongbuk 38541, Korea; arslan@yu.ac.kr (A.M.); zulqarnain@yu.ac.kr (Z.N.); yazdan@yu.ac.kr (Y.A.Q.); 2 School of Intelligent Mechatronics Engineering, Sejong University, Seoul 05006, Korea; rashidali@sejong.ac.kr

**Keywords:** Internet of Things, IEEE 802.15.4, MAC protocols, RPL, reinforcement learning, Q-learning

## Abstract

The next generation of the Internet of Things (IoT) networks is expected to handle a massive scale of sensor deployment with radically heterogeneous traffic applications, which leads to a congested network, calling for new mechanisms to improve network efficiency. Existing protocols are based on simple heuristics mechanisms, whereas the probability of collision is still one of the significant challenges of future IoT networks. The medium access control layer of IEEE 802.15.4 uses a distributed coordination function to determine the efficiency of accessing wireless channels in IoT networks. Similarly, the network layer uses a ranking mechanism to route the packets. The objective of this study was to intelligently utilize the cooperation of multiple communication layers in an IoT network. Recently, Q-learning (QL), a machine learning algorithm, has emerged to solve learning problems in energy and computational-constrained sensor devices. Therefore, we present a QL-based intelligent collision probability inference algorithm to optimize the performance of sensor nodes by utilizing channel collision probability and network layer ranking states with the help of an accumulated reward function. The simulation results showed that the proposed scheme achieved a higher packet reception ratio, produces significantly lower control overheads, and consumed less energy compared to current state-of-the-art mechanisms.

## 1. Introduction

The Internet of Things (IoT) is a promising communication technology that can provide connectivity to physical objects anywhere and anytime. The *things* in IoT refer to sensors, actuators, and microprocessor-based embedded devices [1]. The IoT network consists of a large number of sensors and actuators which are battery-powered and contain limited processing and storage capacity [2]. Internet of Things-based systems have numerous applications such as smart cities [3], smart healthcare [4,5], smart industries [6], and smart grids [7]. Thus, a large number of IoT sensors are expected to be deployed over wireless links in the future. These large-scale networks must be efficient and reliable. For example, in the case of industrial automation, machine-to-machine communication poses new challenges in autonomous data acquisition. Some studies have investigated the incentive mechanism design and cross-layer resource allocation approach [8]. The sensors are expected to be deployed in a complicated environment. The system cost is high, and deployment in a harsh environment is challenging. Therefore, these tiny devices must be able to handle data processing, packet transmission, and energy consumption intelligently. The medium access control (MAC) protocol and routing mechanism can be prescribed with simple mathematical models; however, it still requires a complex protocol. Because the IoT-based network is constrained in terms of its resources, designing an intelligent communication protocol is a challenging task. 

The routing protocol for low-power and lossy networks (RPL) was proposed by the Internet Engineering Task Force (IETF) [9]. The RPL is a de facto routing protocol for resource-constrained IoT devices and is based on the Internet Protocol version 6 (IPv6) low-power wireless personal area network. In the RPL mechanism, the routes are constructed as soon as the network is initialized, indicating that it is a proactive routing protocol. The nodes utilizing the RPL create a tree-like routing topology called a destination-oriented directed acyclic graph (DODAG). The packets are directed to one or more sink nodes, hence the name “destination-oriented”. These routes are created based on some specific objective function (OF). The IETF proposed the minimum rank with hysteresis OF (MRHOF) [10] which is based on expected transmission counts (ETX) and OF zero (OF0) [11], which is based on hop counts, as default routing metrics. The routes in MRHOF are based on the link cost associated with the routes. The link cost or link quality is calculated by broadcasting probe packets at time intervals. The receiving node rebroadcasts the probe packet. This mechanism of continuous link assessment causes congestion in the network. 

Similarly, at the MAC layer, the devices use carrier-sense multiple access with collision avoidance (CSMA/CA) to access the channel [12]. Thus, the device manages the resources dynamically at the MAC layer to proficiently increase the network performance. The overall network quality can be improved by enhancing the capability of the device at the lower layers of the open systems interconnection (OSI) model. Similarly, the network performance can be improved by enhancing the device capabilities to learn traffic heterogeneity and diversity [13]. Thus, cross-layer optimization along with intelligent communication protocols are key to attaining optimal performance in heterogeneous data traffic scenarios. The CSMA/CA mechanism avoids collisions in the network. The collision probability on a wireless channel mainly depends on the number of neighboring nodes in the vicinity. As the network becomes denser, the probability of collision increases and network performance becomes poorer [14].

Moreover, the probability of collision increases with an increase in the network traffic flow. In a real network deployment, the traffic is saturated and heterogeneous. Heterogeneous refers to a lack of uniformity and saturated means that every node always has a packet to send. In the IoT network, some of the nodes may be traffic-intensive, whereas others produce traffic with a low generation rate. Thus, the overall resulting traffic pattern is unpredictable, and load imbalance may occur frequently. However, the current communication protocols lack the adaptability to the heterogeneous data traffic environment and suffer from severe congestion and packet loss due to the lower utilization of the full network capacity. Hence, the devices need an efficient mechanism at both the MAC and network layers to manage the network during fluctuating transmission loads.

Future IoT networks are predicted to have diverse and exciting features that optimize network performance and communication efficiently. Machine learning (ML) is one of the most powerful artificial intelligence tools that provide machines with the ability to learn without a specific program [15]. Machine learning techniques to enable machine intelligence capabilities in IoT communication technologies are attracting much attention [16]. The popularity of ML is due to the fact of its successful application in the area of speech recognition, big data analytics, and language processing.

In the case of industrial automation, machine-to-machine communication poses new challenges in autonomous data acquisition. Some studies have investigated the incentive mechanism design and cross-layer resource allocation approach. Similarly, ML-based edge computing is playing an important role to support the industrial IoT. For example, edge computing, along with a learning-based channel selection mechanism, can achieve high performance for industrial IoT [17]. Popular technology companies, such as Google, Microsoft, and Facebook, are already employing ML-based algorithms to enhance their operational capabilities. The ML establishes a paradigm to learn from data patterns and sequences of actions. It observes the environment and develops an action policy. In the context of IoT networks, an intelligent IoT device would observe and learn from a series of actions to improve a specific objective function. Based on the learning, the device improves its performance by executing future actions and exploiting previous experiences. The ML can be used extensively for improving numerous practical problems in IoT networks [18].

The ML at the routing layer and MAC layer is one of the technology drivers for next-generation low power networks. Similarly, heuristic models are also prevalent. The MAC layer and network layer capabilities for future dense IoT networks can be improved with ML-based algorithms. We utilized a cross-layer approach as a technology driver for a low-power network. Reinforcement learning (RL) is one of the significant ML techniques that are capable of training an agent (IoT device) to interact with an environment to maximize the cumulative reward [19]. For example, the conventional routing procedure involves an exchange of expensive (in terms of computational and energy cost) control packets. The cooperation of multiple communication layers can improve the quality of service. The RL can extract essential correlations between the MAC layer and network layer parameters to learn the network dynamics. Hence, the sensors can handle the communication task independently with reduced resource utilization.

In the IoT-based network, the network layer protocol takes the decisions using a specific OF. For example, the MRHOF is based on the ETX mechanism, and the OF0 applies hop counts to route packets. In the ETX mechanism, the nodes estimate the link quality using a probe broadcasting mechanism at time intervals. The broadcasting and rebroadcasting of the probe packet increase network congestion. Similarly, the queue utilization-based algorithm (QU-RPL) uses queue information along with hop counts and ETX to improve network performance [20]. The stability-aware load balancing (SL-RPL) [21] mechanism is also proposed to strengthen the RPL-based network. The SL-RPL uses packet transmission rate along ETX for a routing metric. The main objective of SL-RPL is to mitigate the frequent parent changing and to provide load balancing. Instead of utilizing the ETX-based mechanism, it is more efficient to exploit the probability of collision information at the MAC layer to learn the network dynamics and consequently improve the overall network performance.

The self-sustainability of low-power and lossy IoT nodes, according to network condition, is one of the open issues of the IoT network. To solve this issue, we present a Q-learning (QL)-based intelligent collision probability learning algorithm (*i*CPLA). The proposed method exploits the probability of collision at the MAC layer to learn the collision in the network. In *i*CPLA, each node recursively examines the environment. In a heterogeneous traffic environment, the probability of collision varies due to the fluctuating traffic flow. However, we can learn and exploit the probability of collision information at the MAC layer to make efficient network layer decisions. 

In *i*CPLA, each node learns the channel collision probability and uses this information at the network layer. In this way, it tunes the RPL-based network layer using MAC layer collision information through interacting with the environment. The *i*CPLA algorithm runs on each node independently; in this distributed way, the nodes update the routing table entries accordingly. The proposed method is an intelligent adaptive protocol that can achieve a defined goal by learning the dynamics of a sensor network. The QL-based mechanism allows sensor nodes to learn to find an optimal forwarding path. In the proposed mechanism, the node determines the different states and performs actions based on the corresponding reward for each action. By updating the Q-values at each iteration, each device makes rational decisions based on its state–action–reward tuple. 

Significant contributions of this study are as follows;

- The incorporation of a cross-layer mechanism is introduced to be used for low-power and lossy networks (LLNs). For the utilization of the cross-layer method, the IPv6 neighbor discovery function, as specified in RFC 4861 is used to calculate collision probability at IEEE 802.15.4 CSMA/CA-based MAC layer.
- A collision probability-based ranking mechanism is developed to construct the routing table entries.
- The learning estimate is updated by exploring and exploiting the environment. During exploitation, control overheads transmissions are reduced, which further improves the energy consumption of nodes. The nodes trickle timer operation is enhanced by suppressing DIO control packet transmissions during the exploitation phase.
- The standard and extended performance assessment metrics (i.e., packets reception ratio, control overheads, and energy consumption during different stages of communication) have been utilized comprehensively to evaluate the performance of the proposed mechanism.

The rest of the paper is structured as follows. Section 2 discusses ML for IoT-based systems. Section 3 presents the proposed *i*CPLA mechanism in detail. Section 4 demonstrates the performance evaluation of the proposed mechanism, and, finally, Section 5 provides a general conclusion along with future research directions.

## 2. Machine Learning for IoT-Based Systems 

The ML algorithms are usually categorized as supervised learning, unsupervised learning, and RL. In supervised learning, the agent learns the history to map the input and output data for future predictions. The agent has input and output variables and determines the mapping function from the input to the output. Thus, the agent can predict the output variables using the input data. The supervised learning algorithm trains an agent to generate a response to a new dataset. The objective of supervised learning is to find relationships in the input data to effectively generate the output data for future data processing [22]. Predictive models are developed using classification and regression techniques. The supervised algorithms include linear regression [23], k-nearest neighbor [24], support vector machines [25], and Bayesian learning [26]. The supervised learning algorithms are significant ML techniques; however, they are not suitable for systems where an agent requires learning without the help of a supervisor and training dataset.

By contrast, in unsupervised ML, only the input data are available. The data in an unsupervised system are unlabeled and uncategorized. The agent deduces information or patterns from an input dataset without reference to a known outcome. The goal is to find symmetries in the dataset to categorize it into groups or clusters for better understanding. The unsupervised algorithms include principal component analysis, k-means clustering, and independent component analysis. Unsupervised ML helps to find features and known patterns in data. The main applications of unsupervised ML techniques include anomaly detection and data processing to reduce the number of features in the dataset [15]. Similarly, the effect of ML for securing the data and network information is very profound [27,28]. 

The RL concerns how the system or agent takes action in an environment to obtain the optimal reward. The aim is to find the most efficient method of accomplishing a particular goal. In a sensor network, the sensor node is the learner and a decision maker. Several factors influence the decision to select a specific action in a particular environment. The node seeks to find the best action for each possible input; thus, it iteratively learns the actions to pursue its goal, adapting to various network conditions [19].

In learning algorithms, the agent learns the environment and then performs the actions. During learning, it explores different actions called the exploration phase. If it selects the best performing action, it is called exploitation. The multi-armed bandit (MAB) is an RL decision-making probability-solving technique to trade-off exploration and exploitation. Exploitation means choosing the best decision given the current information, and exploration denotes gathering more information [29]. In RL, there are *k* possible actions that a sensor node can take. For example, in a tree-like topology forwarding mechanism, a child node selects a parent node from *N* candidate parent nodes; this is the action of a child node. In return, the child node receives a reward. In the next period, the selection (i.e., whether the child node should continue using that parent node (i.e., exploitation) to deliver the packets to the sink node or it should select a new parent (i.e., exploration)) is solved via the MAB technique. Balancing exploration and exploitation is a challenging task in RL because too much exploration may yield negative rewards, and too much exploitation of a particular environment may result in preventing optimal long-term rewards.

The epsilon-greedy (∈-greedy) MAB technique is used to balance exploration and exploitation [30]. With the ∈-greedy technique, the sensor node selects the best available option; however, with every occasion, it also explores the other available options with a probability of 0≤ ∈ ≤ 1. A value of ∈ = 1 means that the algorithm always chooses a random action. Similarly, ∈ = 0.7 gives 70% time of exploration and 30% time of exploitation. Thus, we can add randomness to the learning algorithm by varying the ∈ value.

Different network layer approaches have been proposed to improve the performance of IoT-based network. To enhance the quality of the link, Ancillotti et al. [31] proposed an RL-based link quality estimation (LQE) strategy for RPL. It measures the trend of the received signal strength indicator (RSSI) and uses the RSSI value along with the ETX metric. This method improves the RPL network link repair procedure but increases the control overheads. Similarly, Aziz et al. [32] used a MAB-based clustering method for the probing system of the ETX metric. This method uses a clustering technique to optimize the network. Communicating with cluster heads increases the control overhead. Non-ML techniques are also proposed to improve IoT network communication. For example, Tang et al. [33] discuss the congestion avoidance mechanism and proposed a composite metric named CA-RPL. The CA-RPL computes the weight of each path. It also includes ETX value alongside the number of received packets. The utilization of the ETX metric in this method also increases the control overheads. Another method, CoA-OF, uses ETX, QU, and residual energy metric for parent selection [34]. It improves packets delivery ratio, energy consumption, and throughput, but it introduces frequent parent changes in high traffic scenarios that also result in increased control overhead.

Enhanced RPL (E-RPL) is proposed to decrease the control overheads [35]. This method limits the nodes to wait for the control packet to update rank information. Limiting control packets improves energy consumption; however, it increases the network convergence time. The fuzzy logic-based mechanism is based on ETX and energy consumption for rank calculation [36]. It shows improvement in the network throughput but increases overall energy consumption. Ghaleb et al. [37] proposed a solution to balance the load by monitoring the list of child nodes and also introduced a fast propagation timer to update the child list. The proposed method improves the packet delivery ratio and energy consumption, but it presents a looping in the network which increases the convergence time. The genetic algorithm-based method is also proposed which utilizes weighted queue length, delay, residual energy, ETX, and hop counts metrics [38]. The algorithm improves the packet’s average success ratio, end-to-end delay, and remaining energy. However, the network control overheads are not considered in this research. Taghizadeh et al. [39] utilized the QU factor to solve energy and packet loss problems under heavy traffic load scenarios. The proposed scheme improves energy consumption and packet loss ratio. However, this method also increases the control overheads that updates the node rank information. 

Next-generation technologies must shift from rule-based protocols to learning-based methods to improve the network conditions. The intelligent, self-sustaining IoT nodes are the key to sustainable smart IoT applications. So, integrating learning capabilities according to environmental conditions can support extensively advanced applications for smart cities.

## 3. Proposed Reinforcement Learning-Enabled Optimization of Low-Power and Lossy Networks (LLNs)

In this section, we present the proposed RL-enabled optimization of LLNs. This section is divided into four subsections. The first subsection explains the reinforcement learning and Q-learning model. The second subsection describes the IEEE 802.15.4 collision resolution and path forwarding mechanism, whereas the third subsection explains the problem formulation and system model. Finally, the fourth subsection provides an intelligent QL-based algorithm to optimize the performance of LLNs.

### 3.1. Reinforcement Learning and Q-Learning Model

In RL, the device learns the actions required to map the situations for these actions in the environment. The RL-based algorithms are usually based on an estimated value function (state–action pairs) which indicates whether the current state is good or bad. The objective of the RL-based algorithm is to maximize the numerical reward. The trade-off between exploration and exploitation is fundamental in the RL algorithm. The devices should explore actions that provide global optimum solutions. The purpose of each action is to acquire the maximum reward. At the start, it is possible to find actions that can lead to poor performance. Thus, in stochastic tasks, each action must be tested several times to gain a consistent estimation of its expected reward [19]. The RL system has four main sub-elements: a policy, a reward signal, a value function, and sometimes (optionally) an environment model.

The QL technique is a model-free RL technique for solving decision problems. The “Q” in QL stands for quality. The quality shows how useful the current action is in obtaining a high reward. The QL algorithm creates a Q-table of states and actions. After each episode, the table is updated, and the device then chooses the best action based on the Q-value. The Q-value is updated based on the learning rate, discount factor, the difference between the discounted new value and old values (ΔQ), and reward. The learning rate is represented by α which indicates the extent to which the new value overrides the previous one. 

**Figure 1:**
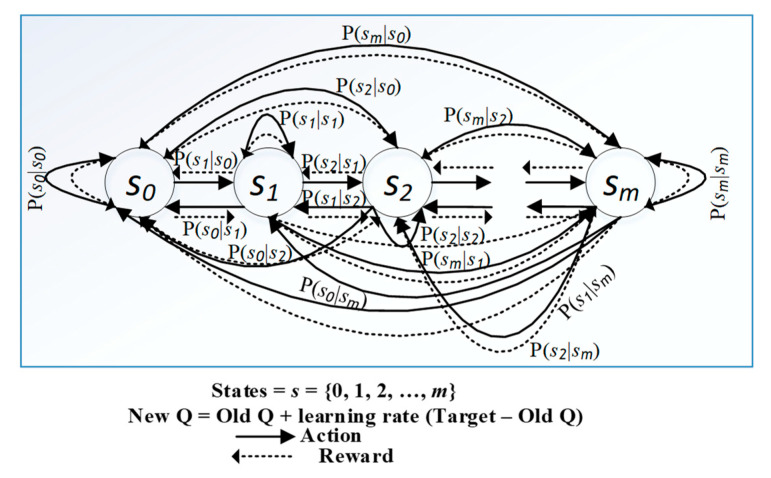
State-transition diagram for Markov decision process (MDP) with *m* states.

The sensor node decides to select an action under a particular state depending on the policy π(a|s)*,* also referred to as policy function. A Bellman equation determines the optimal policy and value function, which allows us to formulate an equation that represents our state–value function [19] as shown below:

(1) $$Q^{\pi *}\left(s_{t},\;a_{t}\right)=E\{r_{t}+\beta \times max_{a^{\prime }}Q^{\pi *}\left(s^{\prime },a^{\prime }\right)|s_{t}=s,\;a_{t}=a\},$$

where β represents a discount factor (0 ≤ β≤ 1), which affects how much weight an algorithm gives to future rewards in the value function. A discount factor of β = 0 results in a state–action value representing the immediate reward, while a higher discount factor of β = 1 results in a state–action value representing the cumulative discounted future reward. The $r_{t}$ represents the reward of the action $a_{t}$ at time t. There are multiple possible actions determined by a policy π(a|s). Each possible action is associated with an action–value function $Q^{\pi }\left(s,\;a\right)$, returning a Q-value of that particular action. In QL, Q(s, a) estimates the reward as the aggregated reward, and it is updated using the following equation:

(2) Q(s, a)=(1−α)×Q(s,a)+α×ΔQ(s,a),

where α is the learning rate with a value between 0 and 1, which indicates the extent to which the new value overrides the previous value. As shown in the equation, if α = 0, the device does not learn a new value, whereas if α = 1, the device only considers a new value. Hence, for a higher learning rate, the learning estimate fluctuates because, in each episode, the node gives more consideration to new value irrespective of its previous experience. In the above equation, ΔQ(s,a) is an improved learning estimate defined as follows:

(3) $$\Delta Q\left(s,a\right)=\left\{r\left(s,a\right)+\beta \times min_{a}Q\left(s^{\prime },a^{\prime }\right)\right\}-Q\left(s,a\right),\;$$

where β indicates if the current reward is more desirable than future rewards. The $min_{a}Q\left(s^{\prime },a^{\prime }\right)$ is the minimum (best) Q-value obtained from the state–action pair. The QL gives the wireless sensor node an ability to discover its reward and new state from the environment and makes it applicable for optimizing the LLN devices. The mathematical framework to describe states s, actions a, reward r, and state-transition probabilities P is shown in Figure 1. The greedy action can be written as:

(4) $$a^{\pi }=argmin_{a}Q\left(s,a\right),$$

where $argmin_{a}$ represents the exploitation of Q(s,a) concerning action a. The continuous exploration in a greedy manner increases the instant reward. As the number of episodes increases, the value of Q(s,a) moves toward the optimum value of $Q^{*}\left(s,a\right)$.

### 3.2. IEEE 802.15.4 Collision Resolution and Path Forwarding Mechanism

The IEEE 802.15.4 standard specifies the MAC layer and the physical (PHY) layer for LLNs [40]. It utilizes the CSMA/CA mechanism to access a wireless channel. The MAC layer uses a distributed coordination function (DCF) to access the channel [41]. Similarly, LLN nodes utilize the RPL method for the path forwarding mechanism.

#### 3.2.1. IEEE 802.15.4 CSMA/CA BEB Mechanism 

The CSMA/CA protocol is based on a DCF mechanism with a binary exponential backoff (BEB) algorithm for resource allocation. The objective of the CSMA/CA BEB protocol is to minimize collisions due to the fact of multiple nodes simultaneously accessing a wireless medium. In the DCF mechanism, when a node has a packet to transmit, it first listens to the channel status for a short duration, called the DCF interframe space (DIFS) interval, to determine if the channel is idle or in use. If the channel is empty for a DIFS duration, the node proceeds to transmit the packet. If the medium is occupied, the node postpones its transmission until the end of the ongoing communication. The node initializes its backoff timer with randomly selected backoff intervals from 0 to *CW_i_−1*, where *CW_i_* is the current contention window size, and *i* is the backoff stage. The BEB counter decrements again every time the channel becomes idle for a DIFS. The probability of two or more nodes selecting the same backoff value is low [42]. The size of *CW* depends on the number of failed transmissions due to the fact of collisions. At the first collision, the size of *CW* is *CW_min_*; after each collision, *CW* is doubled until it reaches the maximum value (*CW_max_*). The *CW* remains at the maximum value until a successful transmission or when it reaches the maximum retransmission limit [43].

#### 3.2.2. RPL Routing

**Figure 2:**
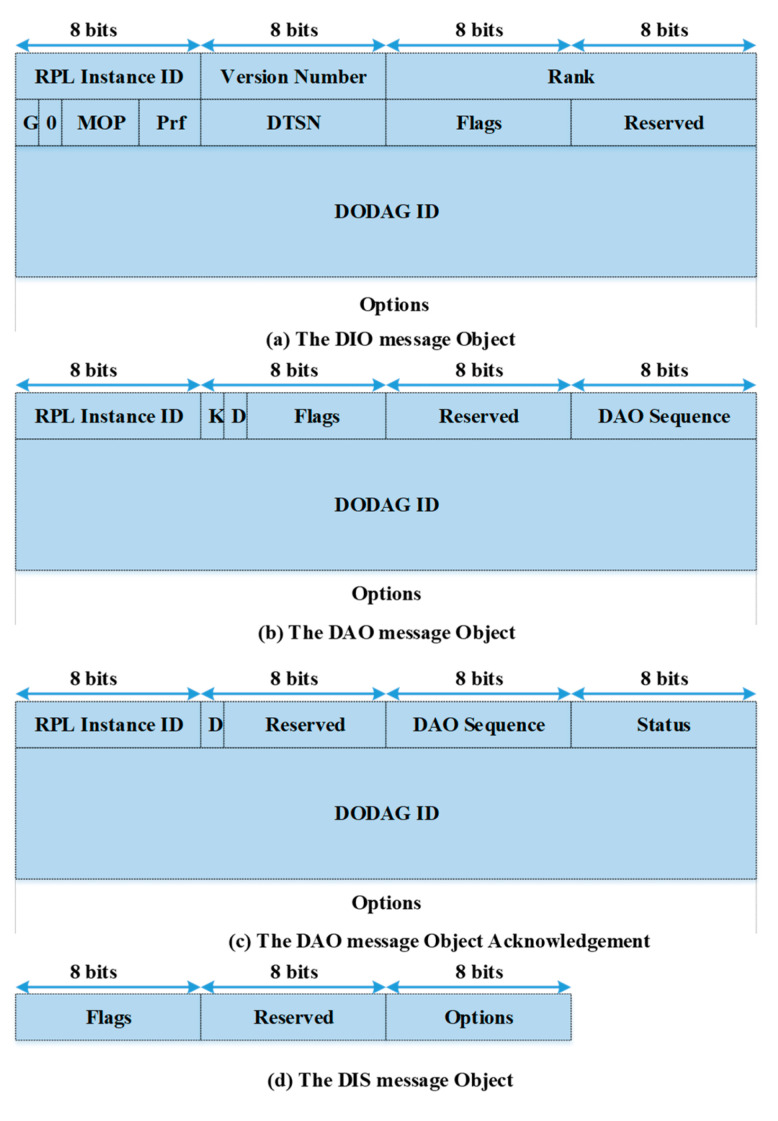
Formats of RPL control messages.

The RPL uses a ranking mechanism as a conventional route selection method. In the RPL, the route construction involves an exchange of control messages, namely, destination oriented directed acyclic graph (DODAG) information objects (DIO), destination advertisement objects (DAO), destination advertisement object acknowledgments, and DODAG information solicitations (DIS) [9]. These messages are very crucial for optimizing network performance. The format of these control messages is shown in Figure 2. The transmission of control message DIO is based on a timer selected by an algorithm called the trickle timer algorithm [44,45]. The DIO transmission rate increases exponentially if the network is inconsistent but decreases to its initial rate if the network is consistent. For a system with a heavy traffic load, the DIO messages result in more congestion and poor network performance. They also cause unnecessary network delays. The two objective functions (i.e., OF0 and MRHOF) for routing decisions in the RPL perform poorly. The OF0 selects the forwarding path based on a simple hop-count irrespective of the path condition, whereas the MRHOF assesses the link quality using a probing method and introduces overheads. 

### 3.3. Problem Formulation and System Model 

The IoT networks have wide application areas, including smart, sustainable cities. The sensor nodes transmit their data to the sink or root node via multiple relay nodes. Each sensor sense and produce data according to its application requirements. Thus, the overall resulting traffic pattern is unpredictable, and load imbalance may occur frequently. The internet layer defines the path, rules, and regulations for the LLN nodes to transmit the data to the sink node. The LLN nodes often suffer from packet loss due to the fact of collision in the densely deployed network. Future generation technologies are expected to be more intelligent, self-sustaining, and adaptive. By integrating the learning capabilities in the LLN devices, the system can be more efficient and self-sustainable. The overall network efficiency can be improved by enhancing the capabilities of the device at lower layers of the OSI model to achieve this goal.

In this study, we utilized a QL technique. The agent was a sensor node, and the environment was the wireless medium. The sensor node learns the environment based on the value function. The function evaluates how good the action is in a given state. The system must satisfy the Markov property called the Markov decision process (MDP) to apply the RL to provide learning and intelligence [46]. The MDP is a mathematical framework for modeling decision making in a specific environment as illustrated in Figure 1. Figure 1 represents the state-transition diagram of the MDP of our proposed system. This figure depicts that a node transmits its state by selecting a forwarding node and updates its routing entries as well as rank information in a practical case study, such as a smart city or smart grid, where a system includes hundreds of connected sensor nodes. In such a system, a transmitting node is always searching for an efficient forwarding neighbor node. The probability that the agent moves into its new state $s^{\prime }$ is known as state-transition probability $P\left(s^{\prime }|s,\;a\right).$ P is the probability of the agent making action a at state s and moving to the new state $s^{\prime }$. Moving from one state to another requires an action space such as,

(5) $$\sum_{s^{\prime }\in S}P\left(s^{\prime },\;r|s,\;a\right)=1,\;\mathrm{for}\;\mathrm{all}\;s\in S,\;a\in A\left(s\right).$$

which shows that the sum of the probabilities of taking *n* number of actions is 1. For example, during path selection, the node either decides to switch the parent node or not. If it decides to change, the action of switching has a probability of 0.5. If it does not change, the probability of not switching is also 0.5, which is like that of a simple probability problem of tossing a coin. The reward function gives the reward for acting a from state s and transiting to $s^{\prime }$. In the MDP, the first step is to specify the system state-space and action-space and satisfy the Markov property. One of the primary purposes of the implementation of a QL algorithm is to construct a mathematical framework to solve MDP-based problems. For example, QL Equations (1)–(5) are helpful to measure how good an action is in a state, or it helps us to understand what actions to take under different states. There exist several practical case studies which utilize these equations for system optimization. For example, the authors in Reference [5] proposed a QL-based *CW* optimization mechanism which uses these equations to select a *CW* size optimally. 

In the proposed mechanism, the network is created using a graph called DODAG, which is based on a parent–child topology. There are N=P U C communicating nodes, and each node is ranked based on its position in the graph. Where $P=\left(p_{1},\;p_{2},\;p_{3},\ldots ,\;p_{i}\right)$ represents the set of parent nodes, and $C=\left(c_{1},\;c_{2},\;c_{3},\ldots ,\;c_{j}\right)$ is the set of all child nodes in the network. The root node is denoted as rank 1, the node next to it as rank 2, etc. Each child node forwards its packets to its selected parent node. Each node utilizes the CSMA/CA BEB channel access mechanism for the contention. The nodes are placed randomly with random distances from each other. Some of the nodes produce heavy traffic while others generate packets with low transmission rates. Thus, the traffic load is heterogeneous with fluctuating and unpredictable patterns. The maximum allowable transmission attempts for the link layer are eight retransmissions, and the CSMA/CA maximum backoff exponent is five.

### 3.4. Proposed Intelligent Collision Probability Learning Algorithm 

**Figure 3:**
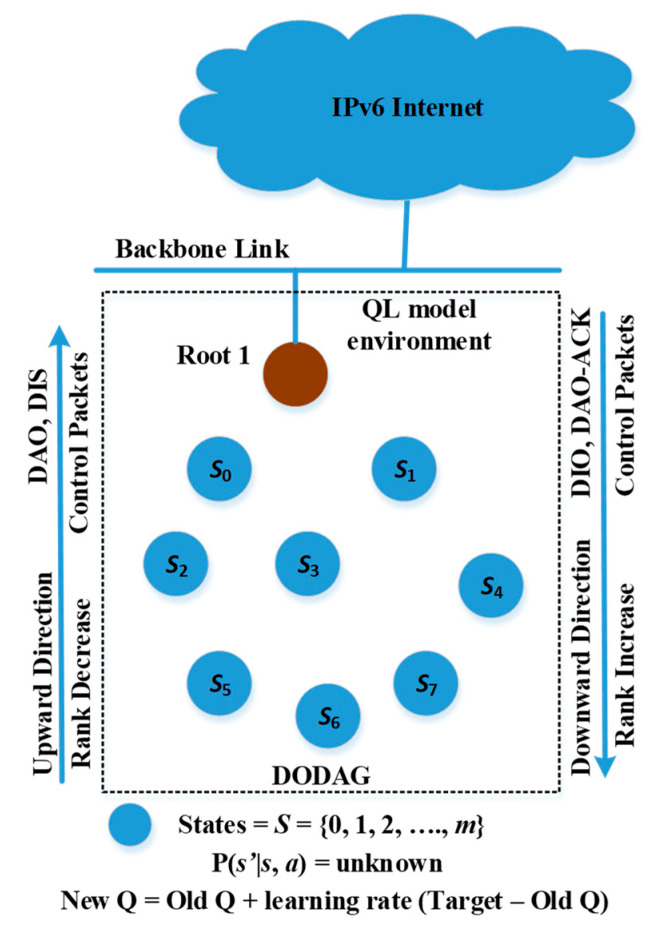
Q-learning model environment for intelligent IoT system device.

In this subsection, we explain our proposed optimized QL-based *i*CPLA mechanism for more efficient LLNs. The QL model and its elements for the proposed mechanism are presented in Figure 3. In the *i*CPLA protocol, we define the state *s* of each node as its neighboring nodes, i.e., *s* ∈ *S* (*S* = {0, 1, 2, …, *m*}), where m denotes the number of states. The value $min_{a}Q\left(s^{\prime },a^{\prime }\right)$ provides the best-estimated value for the potential state *s’*. The selection of a particular neighboring node for path forwarding is the action. The proposed protocol has two-fold changes to the standard one. First, it replaces the ETX mechanism for forwarding path decisions with the CSMA probability of collision. Second, it learns the collision probability from the MAC layer to be used during the exploration and exploitation phases and consequently constructs the routing table entries. 

In wireless communication, MAC protocols rely on the DCF mechanism to sense and assess the channel and avoid channel collision by performing an exponential backoff. In a shared medium, collisions happen when multiple transmissions coincide. The collision probability increases when the network is dense with a saturated traffic load. The theoretical collision usually is derived from the assumption that each node has a saturated traffic load. A saturated node means that the node always has traffic to transmit. 

By estimating the probability of collision in a heterogeneous traffic environment, it is possible to learn the network dynamics. The load imbalance caused by the heterogeneous traffic condition profoundly impacts on the network’s performance due to the collisions. The node increases the backoff exponent (BE) with a range of 0–5 at each collision. The collision probability is defined as a function of the minimum BE stages and contention window size during a given time slot. The node then calculates the *CW* size using the following equation:

(6) $$CW=2^{BE}-1.$$

Using (6), the value of *CW* is in the range of 0–31. The collision probability is defined as at least one of the other *k* − 1 neighbors that transmit simultaneously. Based on the *CW* estimates, the node calculates the probability of collision as follows:

(7) $$P_{coll}=1-{\left(1-1/CW\right)}^{k-1},$$

(8) $$\bar{P_{coll}}=\frac{1}{y}\times \sum_{x=1}^{y}P_{coll\left(x\right)},$$

(9) $$P_{coll}\left(N_{i}\right)=\left(\bar{P_{coll}}+P_{coll\left(current\right)}\right)/2,$$

where *k* represents the number of neighboring nodes, $P_{coll}$ is the probability of collision, $\bar{P_{coll}}$ is the mean probability of total current intervals (i.e., *y*), *x* is a counter for each collision value. *N_i_* represents *i*^th^ sensor node, and $P_{coll}\left(N_{i}\right)$ is the probability of collision of *N_i_* node to be used during the construction of the routing table. The information of the neighboring node is obtained from the network layer. The RPL-based nodes use the DIS control packet to probe its neighborhood. The RPL uses the IPv6 neighbor discovery concept as specified in RFC 4861 [47]. The analytical model in (7) is provided in Reference [48]. Based on the backoff window, the probability of transmission in an arbitrary slot is given by 1/CW. The analytical model in (7) has been widely used in the literature [49]. With each transmission interval, the node calculates the mean of the collision probabilities of the last five intervals as indicated in (8). In (9), the average current collision probability with the prior mean collision probability is obtained. The network layer utilizes this information for exploration during forwarding path selection decisions. The network layer is based on the RPL as described in Section 3.2.2. In the RPL, the routing decisions are made using the rank information embedded in the DIO control packets. Each node broadcasts the DIO packet. The nodes generate a list of potential parent nodes using ranks obtained in the DIO messages. In the RPL method, during the forwarding path selection, the node with the smallest rank is picked as a parent node. In the proposed mechanism, the probability of collision information from the MAC layer is embedded in the DIO message for the rank calculation as follows:

(10) $$Rank\left(c_{j}\right)=Rank\left(p_{i}\right)+P_{coll}\left(p_{i}\right),$$

where $Rank\left(p_{i}\right)$ is the parent node rank, and $P_{coll}\left(p_{i}\right)$ is the collision information obtained using (9). The ETX-based node uses a probe broadcasting mechanism at each interval to measure the quality of links. To alleviate congestion and overhead problems caused by the ETX probing mechanism, we did not utilize the ETX information in the rank calculation. The probability of collision reflects congestion without producing extra overheads. 

Each node generates a routing table entry corresponding to the Q-table value during exploitation. Each node also maintains an estimated rank value for each of its neighbors. The probability of collision in (10) is observed using *CW* size and number of *k* − 1 neighboring nodes. As the network becomes denser, the probability of collision increases and the network performance becomes poorer. Since each node has different *k* − 1 neighbors, the obtained $P_{coll}$ is different for each node. It has been observed that collision probability increases with the increase in traffic load [50]. In the case of heterogeneous traffic patterns, some of the nodes may be traffic-intensive, whereas others produce traffic with a low generation rate. Thus, the overall resulting traffic pattern is unpredictable, and load imbalance may occur frequently. Therefore, in a heterogeneous traffic environment, the probability of collision varies due to the fluctuating traffic flow. The nodes perform exploration using Equation (10) and exploitation using minimum Q-values obtained from Equation (2) to reach an optimal action value. 

One of the most critical aspects of IoT communication is the control overhead. The control overheads are regulated by a mechanism called the trickle timer. Because the LLN nodes have limited computational and energy resources, it is highly advantageous to restrict the transmission of overhead packets at the minimum level. The trickle timer schedules a control message transmission and service discovery. The frequency of control packets is adjusted according to network stability. The trickle timer algorithm schedules the transmission of DIO control packets. The DIO packet carries rank information.

The rank information is required only during the exploration phase. Thus, we reset the trickle timer period for the exploitation phase. During exploitation, the node selects the forwarding node using a Q-table value. In this case, nodes do not require the transmission of DIO messages. Thus, the DIO transmissions are halted. The transmissions of DIO packets start again during the exploration phase. This procedure significantly reduces the number of control overheads in the network without compromising the network performance. The RL-based algorithm updates the network information during the exploration phase and exploits the network during the exploitation phase. Consequently, it mitigates the requirement of hasty DIO transmissions during exploitation. 

The reward in (11) represents the channel collision probability. After a node acts a in state s, it receives a reward indicating how desirable such an action is. A positive reward is given if the current probability of the collision value of the current state is less than its previous collision probability. Similarly, a negative reward is given if the collision probability increases. The reward for each node is given as:

(11) $$r\in \;\left\{ \begin{array}{cc} R^{+}, & if\;collision\;decreases\;\\ R^{-}, & otherwise \end{array}\right..$$

In the state-transition diagram of the *i*CPLA mechanism, (Figure 1), the node moves from one state to another with $R^{+}$ and $R^{-}$ as rewards. The reward in (11) is based on observations. The node learns the collision probabilities to optimize the network performance. We used Equations (6)–(10) in our Q-learning-based proposed algorithm to optimize RPL-based network performance. Equations (6)–(9) are helpful to measure the collision probability within a sensor network. Further, we embedded these calculated values in the rank Equation (10) to formulate our reward function as in Equation (11). The child nodes in a sensor network select its forwarding path using the learned Q-value based on the reward formulated in Equations (6)–(11). Thus, in a smart-city or smart-grid environment, nodes can learn to optimize the network’s performance using Equations (1)–(11). Algorithm 1 describes the steps required to optimize the network using our proposed *i*CPLA mechanism.
**Algorithm 1:** Cross-layer optimization for low-power and lossy networks using *i*CPLA**while** the device is on **do****set** maximum retry limit**set** maximum backoff stages**set***CW_min_* = 0, *CW_max_* = 31**set** current reward = 0, ΔQ (*s*, *a*) = 0, Q(*s*, *a*) = 0BE = MIN (n_collisions, CSMA_MAX_BE)measure *CW* using *BE* in (6)calculate $P_{coll}$ using *CW* in (7)calculate $\bar{P_{coll}}$ using (8)
counter ++ **if** (counter = 5), **then**  counter = 1  $P_{coll}$ = $P_{coll\left(current\right)}$**end if**$P_{coll}\left(N_{i}\right)=Avg\left(\bar{P_{coll}},\;P_{coll\left(current\right)}\right)$**if**$\left(P_{coll\left(current\right)}<\bar{P_{coll}}\right)$, **then** reward = positive**else**reward = negativeupdate reward table for *r* (*s*, *a*)update Q-values table according to (2)pick a random value to explore and exploit**if** (exploit), **then**find $min_{a}Q\left(s^{\prime },a^{\prime }\right)$ IP address**else** (explore)**if** (node = root node), **then**  root rank = 1**end if****if** (parent = null), **then**  rank = max path cost**end if****if** (parent != null), then  $Rank\left(c_{j}\right)=Rank\left(p_{i}\right)+rank_{increase}$  $rank_{increase}$ = ($P_{coll}\left(N_{i}\right)$**end if****if**$(Rank\left(p_{i}\right)\;=0$, **then**  rank = base rank (128)**end if**return MIN $\left(Base\;rank+rank_{increase}\right)$**end while**

## 4. Performance Evaluation 

This section describes the simulation study of the proposed mechanism. The Contiki OS Cooja simulator version 3.0 was used for the simulation analysis [51]. The proposed *i*CPLA scheme was compared with OF0, MRHOF, QU-RPL, and SL-RPL. The network was analyzed in a densely deployed heterogeneous traffic environment, where nodes are placed randomly and packets are generated with different transmission rates. The specific MAC layer and PHY layer parameters utilized during the implementation are provided in Table 1. For the heterogeneous traffic pattern, the transmission interval of the nodes varied with respect to the clock second; for example, 1 packet per second; 1 packet every 2 s; 1 packet every 6 s; and 1 packet every 60 s. The nodes utilized the Zolertia Z1 mote platform [52]. The read-only memory (ROM) size was 96 KB with a payload size of 140 bytes. 

### 4.1. Contiki OS Implementation 

The Contiki OS is a C language-based event-driven operating system that provides IP communication support to LLN devices [53]. It gives an emulator named Cooja, which offers an excellent environment to debug, test, and verify the behavior of networking devices. Cooja consists of a simulation visualizer, simulation timeline, and radio logger for analysis, where each simulation is stored in a simulation configuration (CSC) XML file. To support communication, the Contiki OS provides two networking stacks: uIPv6 and Rime. The uIPv6 endorses the implementation of TCP/IP, UDP, and RPLs for low-end devices containing 8 bit microcontrollers that usually have 64 KB to 256 KB of flash memory and 8 KB to 32 KB of random access memory (RAM).

Similarly, Rime supports a communications stack for low-power radios. ContikiRPL implements the OF in three different modules: (1) a protocol logic module that contains DODAG and parent–child association information; (2) a message-construction and message-parsing module that generates ICMPv6 messages and data structures for the network; and (3) an OF module that provides an OF application program interface. A packet travels through each module, and the uIP layer offers minimum functionality to support the full TCP/IP stack. It allows a maximum IP payload size of 140 bytes. The system parameters are according to standardized protocols [40] and Z1 mote specifications [52]. 

### 4.2. QL Parameter Selection 

**Figure 4:**
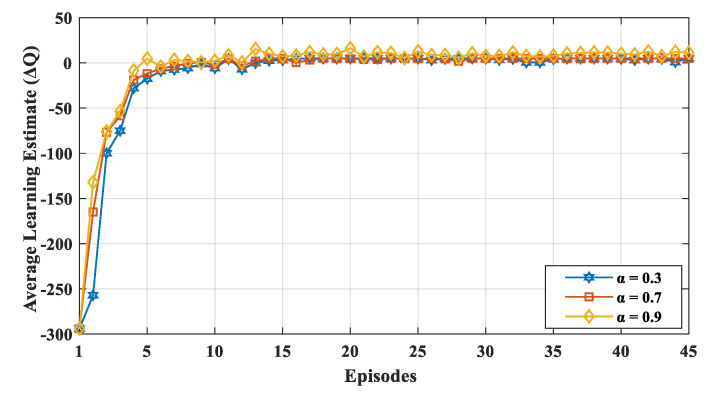
Convergence of learning estimate ΔQ for varying the learning rate α (β = 0.7, ∈ = 0.7).

**Figure 5:**
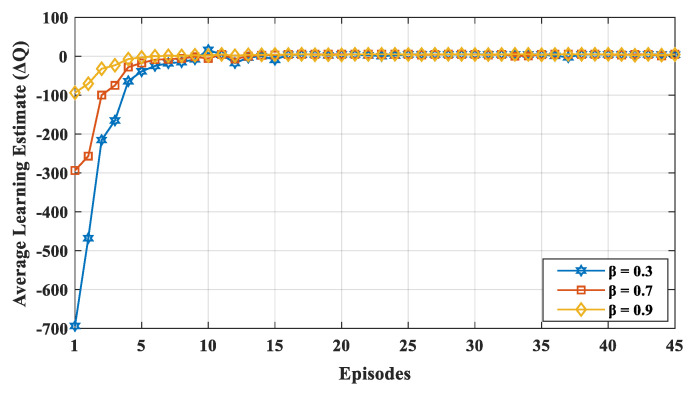
Convergence of learning estimate ΔQ for varying the discount factor β (α = 0.7, ∈ = 0.7).

We evaluated the results with varying learning rate *α* and discount factor β. Similarly, ∈ was varied to learn the network dynamics with different exploration and exploitation values. We varied α and β from small to large values (i.e., 0.3, 0.7, and 0.9) with a probability ∈ of up to 0.7. If the learning rate *α* was high, the learning estimated ΔQ fluctuated, because, in each episode, the node gave more consideration to the new value irrespective of its previous experience. The convergence of the learning estimate ΔQ is depicted in Figure 4 for various learning rates. The value of *α* indicates the extent to which the new value overrides the previous value, and it ranged between 0 to 1. If the value of *α* is closer to zero, the device slowly learns a new value, whereas if the value of *α* is closer to 1, the device only considers a new value and learns faster. Thus, as shown in the Figure 4, for a higher learning rate *α*, the learning estimate ΔQ fluctuated more often, because, in each episode, the node gave more consideration to the new value irrespective of its previous experience. Figure 5 shows the convergence of the learning estimate ΔQ for various discount factors, i.e., β = (0.3, 0.7, and 0.9). The value β affects how much weight it gives to future rewards in the value function. A discount factor close to 0 results in state–action values representing the immediate reward, while a higher discount factor (i.e., β closer to 1) results in the cumulative discounted future reward. The discount factor profoundly influences the convergence of learning estimates. Thus, the value of β is determined based on the convergence of the learning estimate. If a device values the long-term future reward more (i.e., high discount factor β), the learning estimates are closer to the optimal value. If the device gives more value to the current reward, the learning estimate highly diverges from the optimal value initially. The value of ΔQ is obtained using (3). Therefore, a small value of α and substantial value of β makes ΔQ converge faster with less fluctuation. According to Reference [54], Q-learning will converge if the learning rate goes to zero or small state–action space will also converge if the learning rate is low and fixed. In our proposed mechanism, the nodes visit each state–action pair a number of times and updates the process. During the time duration of the network, all state–action pairs are performed.

**Figure 6:**
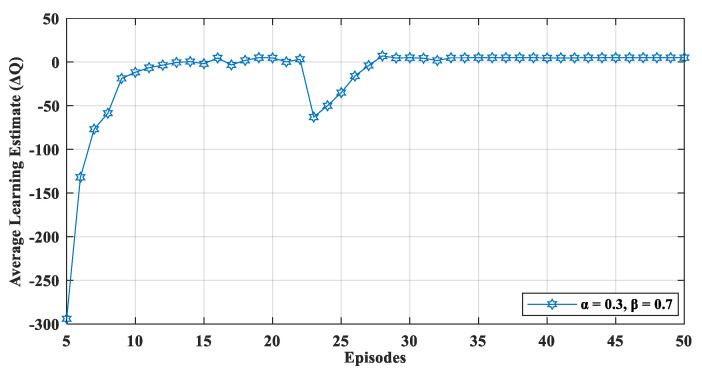
Convergence of learning estimate ΔQ in a dynamic network environment (adding new nodes in the network during simulation).

The convergence of ΔQ indicated that the nodes in the network learned the environment. The nodes exploited the learned information to make better decisions. The ΔQ value was not uniform at the start owing to the exploration of the environment which also means that the value function was not optimized at this stage. With each episode, the nodes tended to move toward the positive reward state. In the early stages of learning, the Q-function was not adequately trained. We can observe that after ten episodes, the network started to become stable which led to the optimization of the performance. Each episode had a 10 s duration of transmission interval. The convergence of ΔQ indicates that there existed an optimal solution for the environment, and the network converged to a stable equilibrium. We also evaluated the learning estimate in a dynamic network environment by adding 20 more nodes to the network during the simulation. Figure 6 displays the effect of a dynamic network environment on ΔQ which shows that after 23 episodes, when new nodes were added in the network, the proposed mechanism performed learning activities to relearn the network dynamics. Therefore, the value of learning estimate (ΔQ) fluctuated when the episode was 23 (Figure 6). Later, the network again converged and, thus, optimized the network’s performance in a dynamic setting. 

### 4.3. Packets Reception Ratio 

**Figure 7:**
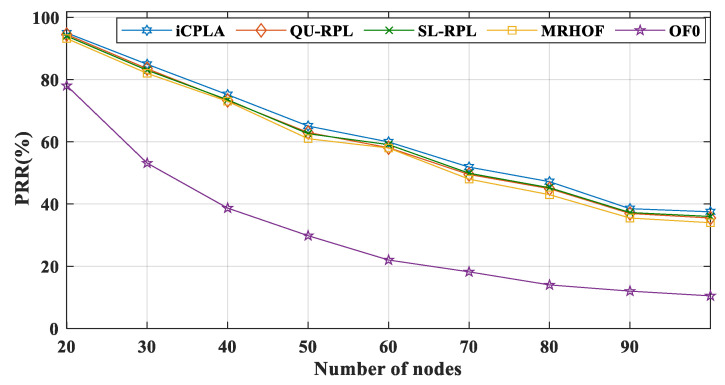
Comparison of packet reception ratio (PRR) between *i*CPLA, QU-RPL, SL-RPL, MRHOF, and OF0 with α = 0.3 and β = 0.7 in a network of 20 to 100 nodes.

The packet reception ratio (PRR) is the total number of packets successfully received by the sink node divided by the total number of transmitted packets. To evaluate the performance of the *i*CPLA, we compared its simulation results with those of the de facto MRHOF, OF0, and recently proposed SL-RPL along with QU-RPL. Figure 7 shows that the *i*CPLA optimized the PRR. Its performance was better than those of the other protocols, indicating that the learning-based protocol was useful for learning the wireless network. The SL-RPL, QU-RPL, and MRHOF also developed a more reliable system compared to the OF0. The OF0 did not contain any link reliability protocol and utilized only the hop-count information. The MRHOF used the ETX-based link assessment method.

Similarly, the QU-RPL was based on hop-counts, ETX, and queue utilization for path selection. The SL-RPL also utilized ETX for routing metrics. The *i*CPLA used the collision probability information from the MAC layer to assess the network’s condition, and the nodes learned the collision information using the QL technique. Thus, it eliminated the need for a computationally expensive ETX mechanism. 

### 4.4. Average End-To-End Packet Delivery Delay 

**Figure 8:**
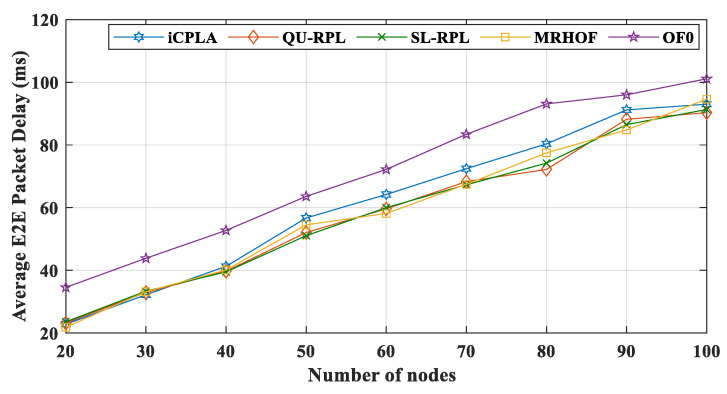
Comparison of average E2E packet delay between *i*CPLA, QU-RPL, SL-RPL, MRHOF, and OF0 with α = 0.3 and β = 0.7 in a network of 20 to 100 nodes.

The average end-to-end packet delivery delay (E2E) shows the average delay of all packets in the network. The E2E delay is the time taken by a packet to reach its destination node from its source. Figure 8 illustrates the performance of the *i*CPLA protocol along with the standard MRHOF, OF0, SL-RPL, and QU-RPL mechanisms in terms of the average E2E packet delay (in milliseconds). The OF0 incurred the highest E2E delay. The OF0 used a forwarding path based on hop-counts irrespective of the congestion status of the links. The MRHOF, QU-RPL, and SL-RPL had almost similar delay patterns, because both they used a continuous probing method. The proposed *i*CPLA mechanism had a higher E2E delay compared to MRHOF and QU-RPL; however, it did not exceed that of OF0. The higher delay was due to the learning process of the intelligent nodes. 

### 4.5. Control Overheads

**Figure 9:**
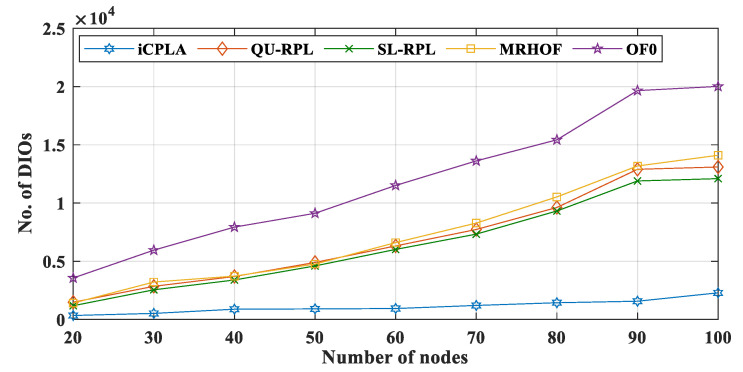
Comparison of number of DIO control messages between *i*CPLA, QU-RPL, SL-RPL, MRHOF, and OF0 with α = 0.3 and β = 0.7 in a network of 20 to 100 nodes.

**Figure 10:**
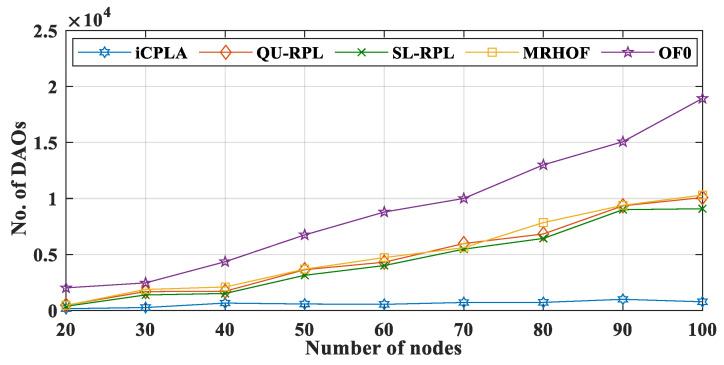
Comparison of number of DAO control messages between *i*CPLA, QU-RPL, SL-RPL, MRHOF, and OF0 with α = 0.3 and β = 0.7 in a network of 20 to 100 nodes.

The IoT devices contain minimal energy, memory, and computational capabilities. The nodes transmit control packets along with data transmission. These control packets significantly reduce the energy, memory, and computational capacity of the devices. Thus, it is imperative to reduce the transmission of control messages without compromising network performance. The RPL incurs three control overheads, i.e., DIO, DAO, and DIS (explained in Section 3.2.2). The nodes maintain the network connectivity using these control packets, and these control packets are regulated by a mechanism called trickle timer. If the network is stable, fewer overheads are exchanged and vice versa. Based on the network conditions, DIO messages are exchanged, and a trickle timer algorithm controls their transmission frequency. Figure 9 and Figure 10 present the total number of DIO and DAO control packets transmitted in the network, respectively. The proposed *i*CPLA method significantly reduced the control packet transmissions compared to QU-RPL, SL-RPL, MRHOF, and OF0. After learning, the ΔQ estimate become stable, and the nodes estimated the collision probability intelligently. The nodes in the *i*CPLA evaluated, learned, and observed their actions based on learning to achieve optimal performance. The network with poor PRR incurred the highest DIO transmission. As an increasing number of packets were dropped, the network became more unstable which led to more DIO transmissions. Therefore, the OF0 incurred the highest DIO overhead in the network. The QU-RPL, SL-RPL, and MRHOF both used the ETX probing method which required more control packets. The QU-RPL incurred slightly fewer overheads compared to the MRHOF due to the utilization of queue information which helped in balancing the load. Similarly, SL-RPL further balances the load by considering the packet transmission rate in its routing metric.

**Figure 11:**
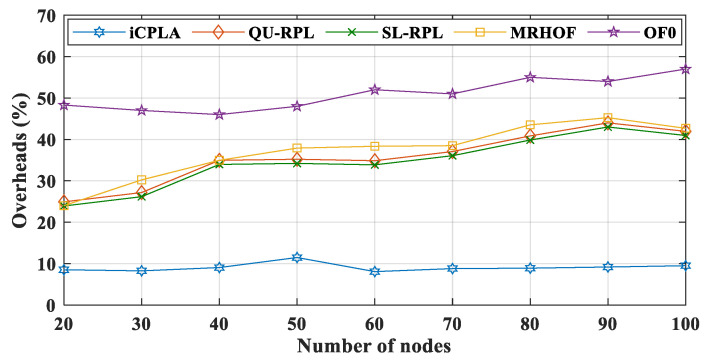
Comparison of total control overhead (%) between *i*CPLA, QU-RPL, SL-RPL, MRHOF, and OF0 with α = 0.3 and β = 0.7 in a network of 20 to 100 nodes.

The destination information of the transmitting nodes was transmitted to the sink node via the DAO control packet. Generally, DAO packets are generated and contribute significantly to the control overhead. The DAO is sent to the sink node when the upward path is changed. With the transmission of DIO and DAO packets, the scarce energy of the nodes is wasted. The proposed *i*CPLA protocol also reduced the number of DAO control packets significantly, compared to the QU-RPL, SL-RPL, MRHOF, and OF0. When the network was initialized, all packets were control messages. The nodes started the transmission of data packets after the network construction was completed. A comparison of the transfer of the total percentage of control overhead versus the data packet transmission is shown in Figure 11. The proposed *i*CPLA protocol had the lowest overall percentage of overhead. More transmission of the control overhead means more energy is wasted in its transmission rather than data packet transmissions. The OF0 had the highest percentage of control overhead, because it was unable to resolve the congestion and load-balancing problem. The *i*CPLA maintained almost the same overhead percentage whether with a small or a large number of nodes. Its total overhead was only approximately 8–9%. The self-learning capability using the RL-based technique gave nodes the ability to make intelligent decisions in an unknown environment. The overhead percentage graphs demonstrate that the *i*CPLA created a consistent, stable, and less congested network which also affected the network’s energy consumption.

### 4.6. Effect on Energy Consumption 

Energy is the most important resource in a sensor network. The IoT nodes consume most of the energy during communication. The nodes spend a significant amount of energy during the transmission and reception of packets. The nodes turn their radios off when they are not transmitting or receiving any packet. The state when the radio is off, and the microcontroller is idle is called low-power mode (LPM). For the Z1 mote, the current consumption during LPM is 20 µA. Similarly, the state when the radio is off, and the microcontroller is on is referred to as CPU idle state (CPUI). The Z1 mote consumes 42.6 µA during the CPUI state. The highest amount of current is consumed during the transmission and reception periods. The transmission current (Tx) consumption is 17.4 mA, whereas the reception current (Rx) consumption is 18.8 mA. These current consumptions come from the Z1 mote specifications [52]. During the simulation, the RTIMER_SECOND value was used to convert the ticks into seconds. The ticks per second value of the Z1 mote was 32,786. During the current consumption of all four stages, a voltage of 3 V was utilized. We measured the energy consumption of the proposed *i*CPLA protocol and compared it to those of the QU-RPL, SL-RPL, MRHOF, and OF0 during all four stages of communication, i.e., LPM, CPU, Tx, and Rx. The energy consumption (in joules) in each state was measured as follows:

(12) LPM=(LPM×0.020×3)/32,768

(13) CPU=(CPU×0.426×3)/32,768

(14) Tx=(Tx×17.4×3)/32,768

(15) Rx=(Rx×18.8×3)/32,768

**Figure 12:**
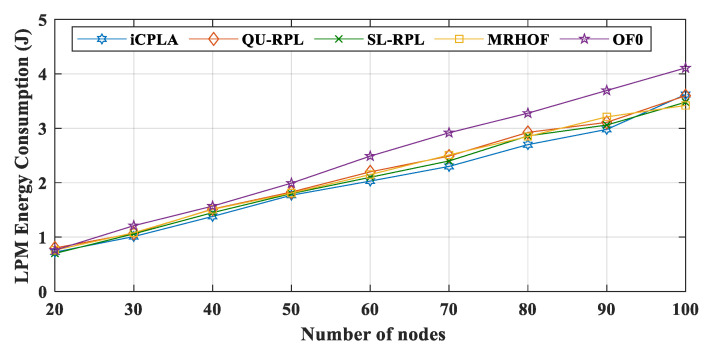
Comparison of LMP energy consumption (J) between *i*CPLA, QU-RPL, SL-RPL, MRHOF, and OF0 with α = 0.3 and β = 0.7 in a network of 20 to 100 nodes.

**Figure 13:**
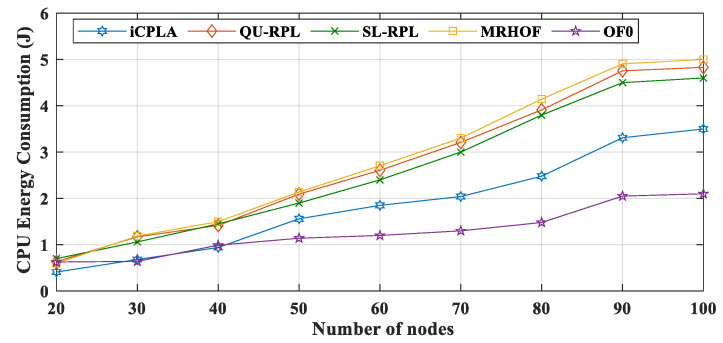
Comparison of CPU energy consumption (J) between *i*CPLA, QU-RPL, SL-RPL, MRHOF, and OF0 with α = 0.3 and β = 0.7 in a network of 20 to 100 nodes.

We utilize the Energest function (*energest_flush()*) in Contiki OS to obtain the LPM, CPU, Tx, and Rx tick values. The LPM energy consumptions of all protocols are depicted in Figure 12. Similarly, the CPU energy consumption comparison between the *i*CPLA, QU-RPL, SL-RPL, MRHOF, and OF0 is exhibited in Figure 13. The MRHOF caused a high CPU utilization due to the calculation of the ETX measurement. During LPM, the sensors conserved energy by turning their radios off. The CPU and LPM energy consumptions were very small, ranging from 0.5 J to 5 J (from small to large networks). The LPM energy consumption was approximately similar in all four protocols. The CPU and LPM energy consumptions were very insignificant compared to the Tx and Rx energy consumptions. 

**Figure 14:**
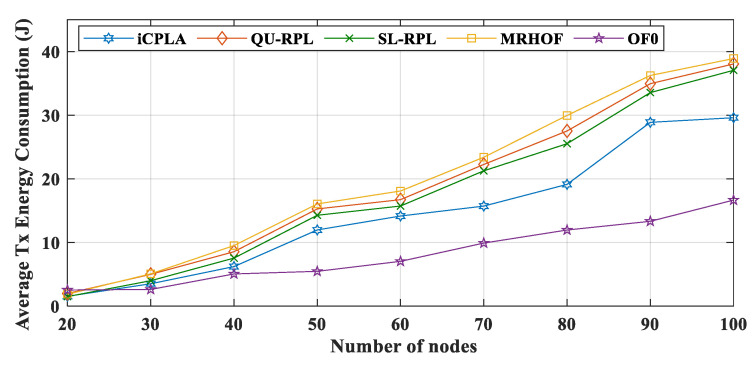
Comparison of average Tx energy consumption (J) between *i*CPLA, QU-RPL, SL-RPL, MRHOF, and OF0 with α = 0.3 and β = 0.7 in a network of 20 to 100 nodes.

**Figure 15:**
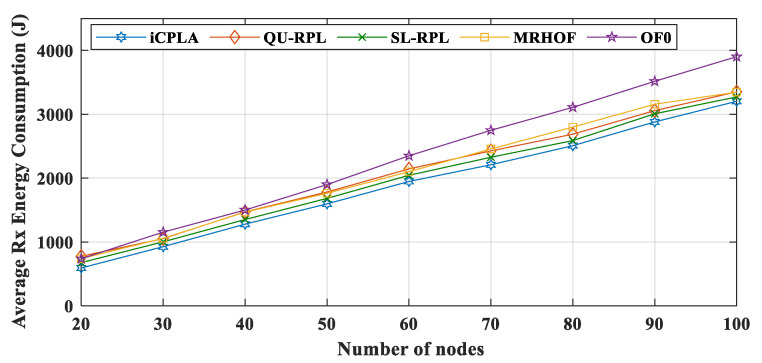
Comparison of average Rx energy consumption (J) between *i*CPLA, QU-RPL, SL-RPL, MRHOF, and OF0 with α = 0.3 and β = 0.7 in a network of 20 to 100 nodes.

The energy consumptions during Tx are displayed in Figure 14. The OF0 had the highest total amount of overhead, but it shows a lower Tx value due to the fact of its poor packet delivery ratio. The size of the data packets was more significant than that of the control overhead; thus, it required more energy for transmission. Because most of the packets were dropped in the OF0, the nodes consumed less energy in transmitting the data packets. The proposed *i*CPLA maintained a Tx lower than those of the QU-RPL, SL-RPL, and MRHOF. The *i*CPLA had the lowest control overhead, and therefore the overall Tx was lower compared to those of the QU-RPL, SL-RPL, and MRHOF. The overhead of *i*CPLA was approximately 8–9% more economical as compared to 20–42% of the QU-RPL, SL-RPL, and MRHOF. Also, it was 48–58% in the OF0, as presented in Figure 11. Similarly, Rx energy consumption is shown in Figure 15. 

**Figure 16:**
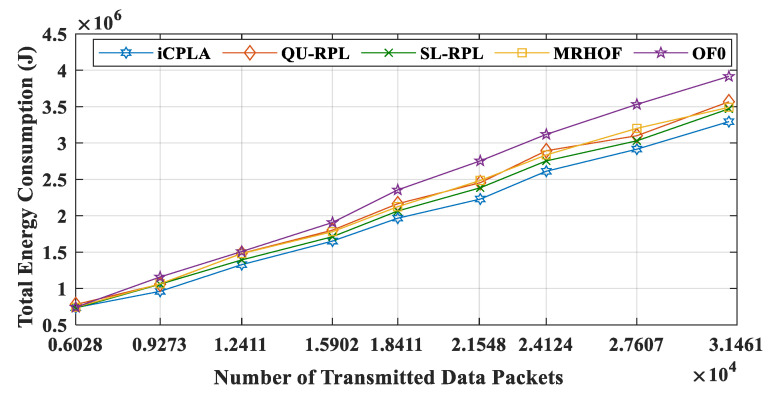
Total energy consumption (J) versus number of transmitted data packets for *i*CPLA, QU-RPL, SL-RPL, MRHOF, and OF0 with α = 0.3 and β = 0.7.

From the results, we can conclude that the OF0 consumed most of the energy for transmitting control packets. In contrast, most of the energy in the *i*CPLA was consumed during data packet transmission. Even with a higher PRR, the *i*CPLA energy consumption was lower due to the significantly lower control overhead. The node spends the highest amount of energy during packet reception. Each node receives packets from multiple nodes and acts as a relay node. The total energy consumption versus the number of transmitted data packets in the network is illustrated in Figure 16. The proposed protocol maintained the lowest total energy consumption compared to the other state-of-the-art mechanisms. Energy reduction is one of the most important design goals in IoT network communication because sensor nodes are required to operate for months or years in a remote location. 

### 4.7. Analysis with Different Network Topologies and Traffic Load Heterogeneity

For further analysis and proof of concept, we also simulated the network with tree topology and grid topology of 50 nodes. The network runs for a longer duration of simulation time (3600 s) with heavy traffic. The traffic heterogeneity is varied concerning clock time. The nodes have high transmission pattern, for example, 2 packets per second; 5 packets per second; 1 packet every 2 s; and 1 packet every 6 s. The performance assessment of tree topology and grid topology with varied heterogeneous traffic load is shown in Table 2 and Table 3, respectively. These results also indicate a better performance assessment of the proposed mechanism in different network topologies.

### 4.8. Computational Complexity 

Reaching a goal state in the RL algorithm requires exploring the entire state space by performing several actions at every state. The numbers of states and actions are finite, and the state space is observable. In RL, every action yields either a positive ($R^{+}$) or negative ($R^{-})$ reward. The reward is obtained after each iteration during the node lifetime. The nodes maintain the Q-value in every state s for every action a, performed in that particular state. The Q(s, a) reflects the total cumulative optimal reward received for every state and action pair. The selection of action is based on a policy that performs its operation in two phases, i.e., exploration and exploitation. The ΔQ is also calculated for every action at a particular state, and utilizing ΔQ in (3) yields the Q-values for a specific state and action pair. Therefore, the computational complexity of the proposed *i*CPLA mechanism is O(n)(a), where n is the number of neighbors (states) that are candidates for being selected as parents, and a is the number of actions available at every state.

### 4.9. Summarization of Results and Discussions

The graphs of the performance evaluation show a significant improvement in the results during the simulation. According to the results, *i*CPLA showed the highest PRR, followed by SL-RPL, QU-RPL, MRHOF, and OF0. The improvement in PRR of the proposed mechanism shows the effectiveness of ML and proves that ML is a promising approach to enhancing network performance in terms of PRR. Similarly, the proposed *i*CPLA method significantly reduced the control packet transmissions compared to other state-of-the-art methods. The trickle timer mechanism can reduce total overheads in the network by intelligently utilizing the ML-based approach as well as optimization. The reduction of overheads and improvement in PRR also affected the total energy consumption of the overall system. The proposed mechanism achieved improved results in terms of total energy consumption which is one of the most important design goals in IoT network communication. The proposed protocol incurred slightly higher delay due to the learning estimation; however, overall, the proposed method showed significantly better performance in terms of packet reception ratio, total percentage of control overheads, and total energy consumption. The proposed *i*CPLA protocol created a consistent, stable, and less congested network environment which means that the proposed protocol has potential in many applications for IoT-based networks. 

### 4.10. Practical Applications of Proposed Framework

**Figure 17:**
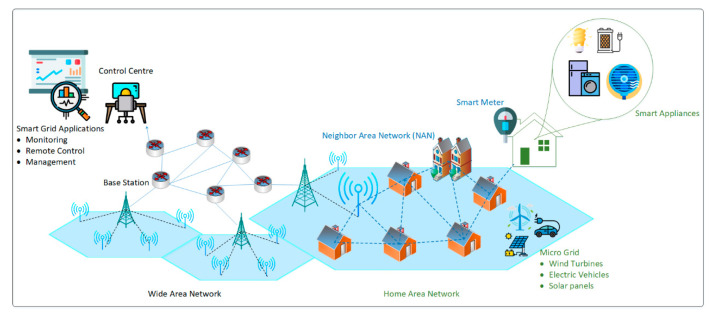
Proposed framework for smart sustainable cities applications.

In the last few years, IoT-based networks and their potential applications have increased exponentially. The IoT networks have given birth to new perspectives on data acquisition and transmission. The IoT networks have the vast potential to affect diverse areas of life, for example, smart, sustainable cities. A smart, sustainable cities’ architecture includes an extensive area network, local area network, home area network, or neighborhood area network. In a smart city, there are several potential applications which include smart parking, smart grids, smart traffic, smart streetlights, public safety, surveillance systems, and so forth. These applications mainly depend on the availability of communication resources including energy resources. The proposed framework for smart, sustainable city applications is shown in Figure 17. We take an example of a smart grid application where numerous IoT devices are involved for critical decision-making purposes. The proposed approach makes use of intelligent cross-layer based MAC and network layer communication to help in accurate delivery of data to the integrated smart grid infrastructure in the smart-cities scenario. As the proposed protocol saves energy, the sensors’ devices would be able to work for a longer duration. According to the proposed scenario, there can be many different types of sensors nodes such as water meters, gas meters, or temperature sensors for weather control. 

## 5. Conclusions and Future Studies 

Futuristic green IoT networks demand an efficient, densely deployed network with heterogeneous traffic applications. Studies on cross-layer optimization for sensor nodes, particularly the impact of heterogeneous traffic on the resource-constrained devices, are still minimal. This paper discussed the handling of collision probability in the dense and congested network. Collision due to the contention-based nature of networks, congestion, and packet loss are the key challenges of future IoT devices. In this paper, the well-known OSI model and particularly the IEEE 802.15.4 and RPL-based network layer were scrutinized to improve the performance of networks. This study presented ML-based algorithms and their applications in the domain of dense IoT networks producing dynamic traffic patterns. Motivated by the promising applications and features of RL for cognitive radios, we presented the utilization of an RL-based intelligent algorithm for densely deployed IoT networks. The proposed learning algorithm uses a smart QL-based method, which is one of the RL techniques, to optimize the performance of LLNs utilizing the cooperation of lower layers of the OSI model. The proposed mechanism learned the collision probability information at the MAC layer to make intelligent decisions at the network layer. The proposed protocol utilized the IPv6 neighbor discovery function as specified in RFC 4861 to calculate collision probability. The method also enhanced the operation of the trickle timer mechanism to reduce the transmission of DIO control packets. The performance of the proposed mechanism was evaluated through extensive simulations using the Contiki 3.0 Cooja simulator. Compared to the de facto standards (i.e., MRHOF and OF0) and QU-RPL and SL-RPL, the proposed scheme offers enhanced performance in terms of PRR, control overhead, and energy consumption. The results indicate the ability of the QL method to enhance the network efficiency in a densely deployed IoT network with heterogeneous traffic. 

In conclusion, ML is a promising area for future wireless research for improving smart application, for example, if we consider the applications of IoT-based smart cities for future generation networks. The main applications could include smart grids, smart parking, smart traffic, smart streetlights, public safety, surveillance systems, etc. 

### Limitations and Future Studies

Currently, we have implemented our proposed framework in a simulated environment. Moreover, due to the learning-based RPL, the computational complexity of the overall system was increased, which is evident in the form of an increased end-to-end delay (E2E). In the future, we plan to address these limitations by implantation of the proposed algorithm in a more realistic environment. We also aim to reduce the E2E delay of the proposed mechanism. In addition, we plan to study an intelligent adaptive trickle timer mechanism to optimize the network further. 

## Figures and Tables

**Table 1:** Simulation Parameters.

Parameters	Value
Contiki OS version	Contiki 3.0
RTIMER_SECOND	32768 ticks/s
Packet size	127 bytes
Simulation time	720 s
Number of nodes	20, 30, 40, 50, 60, 70, 80, 90, 100
PHY & MAC protocol	802.15.4 with CSMA
Packet size	127 bytes
uIP payload buffer size	140 bytes
Buffer occupancy	4 packets
Mote device model	Z1 Zolertia
CW_min_	0
CW_max_	31
Maximum back-off stage	5
Maximum retry limits	8
RAM	8 KB
Flash	92 KB
Tx Current	17.4 mA
Rx Current	18.8 mA
CPU idle current	0.426 mA
CPU power-down current	0.020 mA
Send interval	Varying with respect to clock second.
Script text analysis	Python 3.7

**Table 2:** Analysis of tree topology with varying heterogeneous traffic load.

No. of Nodes	OF	Total Packets Sent	Total Packets Received	Total Packets Lost	PRR %	Packet Lost %	Avg. Delay (ms)	Total Overheads %	Network Energy Consumption (mJ)
50 nodes	OF0	95,126	18,578	76,548	19.52	80.48	56.23	40.25	10,132,887.34
50 nodes	MRHOF	95,318	59,058	36,260	61.95	38.04	48.44	26.47	10,129,675.19
50 nodes	QU-RPL	95,308	60,543	34,765	63.52	36.47	47.95	23.83	10,128,015.73
50 nodes	SL-RPL	95,214	60,973	34,241	64.03	35.97	46.17	22.41	10,121,103.67
50 nodes	*i*CPLA	95,321	62,982	32,339	66.07	33.92	53.67	9.87	10,082,474.88

**Table 3:** Analysis of grid topology with varying heterogeneous traffic load.

No. of Nodes	OF	Total Packets Sent	Total Packets Received	Total Packets Lost	PRR %	Packet Lost %	Avg. Delay (ms)	Total Overheads %	Network Energy Consumption (mJ)
50 nodes	OF0	98,426	18,579	79,847	18.87	81.12	52.71	41.55	10,165,773.69
50 nodes	MRHOF	98,480	57,378	41,102	58.26	41.73	45.98	27.33	10,149,734.52
50 nodes	QU-RPL	98,472	59,288	39,184	60.02	39.98	44.62	25.39	10,140,776.65
50 nodes	SL-RPL	98,467	59,895	38,572	60.08	39.92	44.74	26.12	10,140,102.28
50 nodes	*i*CPLA	98,479	62,018	36,461	62.97	37.02	49.17	10.05	10,133,481.35
